# Cytotoxic Activity of Semi-Synthetic Derivatives of Elatol and Isoobtusol 

**DOI:** 10.3390/md10102254

**Published:** 2012-10-18

**Authors:** Karen L. Lang, Izabella T. Silva, Lara A. Zimmermann, Cíntia Lhullier, Maria V. Mañalich Arana, Jorge A. Palermo, Miriam Falkenberg, Cláudia M. O. Simões, Eloir P. Schenkel, Fernando J. Durán

**Affiliations:** 1 Department of Pharmaceutical Sciences, Federal University of Santa Catarina, Florianópolis, SC, 88040-970, Brazil; Email: izabellathais@hotmail.com (I.T.S.); lalinhazi@gmail.com (L.A.Z.); clhullier@yahoo.com.br (C.L.); miriam@ccs.ufsc.br (M.F.); claudias@reitoria.ufsc.br (C.M.O.S.); eloirschenkel@gmail.com (E.P.S.); 2 Department of Organic Chemistry, University of Buenos Aires, Buenos Aires, C1428EGA, Argentina; Email: vmanalich@qo.fcen.uba.ar (M.V.M.A.); palermo@qo.fcen.uba.ar (J.A.P.); fduran@qo.fcen.uba.ar (F.J.D.)

**Keywords:** elatol, isoobtusol, sesquiterpenes, synthesis, cytotoxic activity

## Abstract

In the present study, the *in vitro* cytotoxic effects of six semi-synthetic derivatives of elatol (**1**) and isoobtusol (**2**) were investigated. Chemical modifications were performed on the hydroxyl groups aiming to get derivatives of different polarity, namely the hemisuccinate, carbamate and sulfamate. The structural elucidation of the new derivatives was based on detailed NMR and MS spectroscopic analyses. The *in vitro* cytotoxicity of compounds **1** to **8 **was evaluated against A459 and RD tumor cell lines with CC_50_ values ranging from 4.93 to 41.53 µM. These results suggest that the structural modifications performed on both compounds could be considered a good strategy to obtain more active derivatives.

## 1. Introduction

The oceanic environment has been a vast source of natural products, yielding a wide range of bioactive compounds with diverse mechanisms of action [1]. The genus *Laurencia* (order Ceremiales, family Rhodomelaceae) is a rich source of halogenated secondary metabolites, predominantly sesquiterpenes, diterpenes, and C15 non-terpenoids, chemically diverse compounds with great therapeutic potential [2].

**Figure 1:**
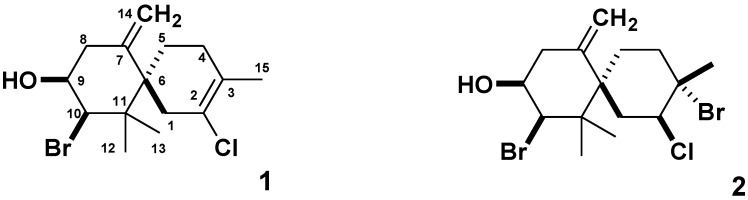
The structure of Elatol (**1**) and Isoobtusol (**2**).

Elatol (**1**) (Figure 1), a halogenated chamigrane sesquiterpene, was isolated for the first time from *Laurencia elata* by Sims *et al*. [3]. Several species of *Laurencia* produce this sesquiterpene as a major secondary metabolite [4,5,6,7], especially *Laurencia microcladia* from which elatol was obtained with the high yield of ca. 10% (w/w) from the ethanolic extract of the alga [8]. This compound has displayed antifeedant [9], antifouling [10], antibacterial [6,11], antifungal [12], antiparasitic [13,14] and cytotoxic activity against HeLa and Hep-2 human carcinoma cell lines [15]. A recent investigation [16] showed that elatol caused *in vitro* an increase in cell numbers at the G1 and the sub-G1 phases, indicating apoptosis induction; and was able to reduce tumor growth *in vivo* in C57Bl6 mice inoculated with B16F10 cells.

Isoobtusol (**2**) (Figure 1) belongs to the same structural class of elatol and was described for the first time from *Laurencia obtusa* by González *et al.* [17]. It showed strong antimicrobial activity against several strains, which included human pathogens [6,12]. 

In spite of this promising pharmacological profile, the high lipophilicity of elatol and isoobtusol and their consequent low aqueous solubility are limiting aspects for further studies. In order to address these issues, chemical modifications were performed on the hydroxyl groups aiming to get analogues with different polarity. With this objective, six compounds were synthetized by reaction with chlorosulfonyl isocyanate and succinic anhydride to obtain the corresponding carbamates (**3** and **6**), sulfamates (**5** and **8**) and hemissuccinates (**4** and **7**). Such derivatives have been shown to improve not only the water solubility, but in some cases also the cytotoxic activity of the compounds, as its amphiphilic character is supposed to enhance absorption in biological systems [18,19]. In this way, these compounds were evaluated for *in vitro* cytotoxic activity against lung (A549) and embryo rhabdomyosarcome (RD) tumor cells, and a preliminary structure-activity relationship (SAR) is presented. 

## 2. Results and Discussion

### 2.1. Chemistry

**Scheme 1:**
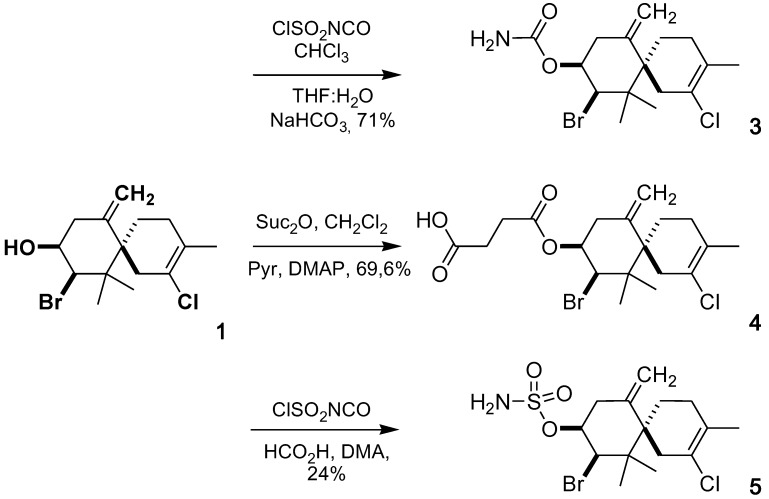
Synthesis of elatol derivatives (**3**), (**4**) and (**5**).

**Scheme 2:**
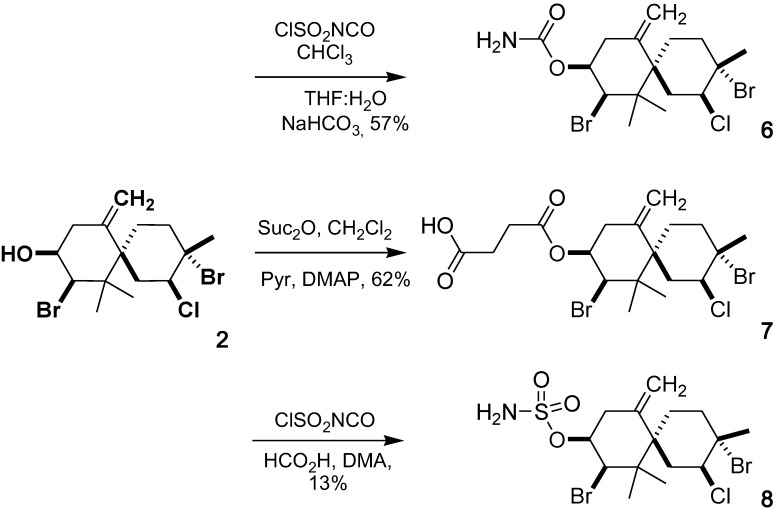
Synthesis of isoobtusol derivatives (**6**), (**7**) and (**8**).

Six derivatives of elatol (**1**) and isoobtusol (**2**) were synthesized by the routes described in Scheme 1 and Scheme 2, and their cytotoxic activity was evaluated.

The carbamate derivative (**3**) was prepared by treatment of (**1**) with chlorosulfonyl isocyanate as described previously by Bandyopadhyay *et al.* [20]. Compound (**4**) was prepared by the reaction of (**1**) with succinic anhydride in presence of DMAP, pyridine and CH_2_Cl_2_. Compound (**5**) was obtained by reaction with chlorosulfonyl isocyanate in the presence of formic acid and DMA [21]. Compounds (**6**), (**7**) and (**8**) were obtained from (**2**) according to the same methods used for the preparation of compounds (**3**), (**4**) and (**5**), respectively. The compounds were purified by silica gel column chromatography with hexane/ethyl acetate as eluant and their structures confirmed by IR, ^1^H-NMR, ^13^C-NMR, 2D-NMR and MS.

### 2.2. Cytotoxic Activity

To quantify the cytotoxic effects of elatol (**1**), isoobtusol (**2**) and their synthetic derivatives (**3**) to (**8**), the CC_50_ value of each compound was measured (CC_50_ is defined as the concentration that reduced cell growth by 50% after 48 h). Table 1 shows the CC_50_ values obtained for the tested compounds against human non-small cell lung tumor (A549) and human embryo rhabdomyosarcome (RD) cells.

**Table 1:** Cytotoxicity of compounds **1** to **8** against two different human tumor cell lines.

Compound	Cell lines [CC_50_^a^ (μM)]	
	A549	RD
**1**	7.56 ± 0.19	11.22 ± 1.63
**2**	14.24 ± 3.43	6.24 ± 1.11
**3**	21.93 ± 1.27	13.26 ± 0.76
**4**	39.05 ± 4.81	21.23 ± 7.16
**5**	>100	41.53 ± 0.36
**6**	39.57 ± 2.07	23.83 ± 5.28
**7**	10.74 ± 2.52	4.93 ± 0.52
**8**	23.85 ± 5.21	20.48 ± 1.44
**Paclitaxel**	0.260 ± 0.027	0.025 ± 0.004

Values represent the mean ± standard deviations of three independent experiments; ^a^ Cytotoxicity was determined by MTT assay on each human tested cancer cell line.

Elatol (**1**) and isoobtusol (**2**) exhibited significant cytotoxicity against the tested human tumor cell lines with CC_50_ values ranging from 6.24 to 14.24 µM. Elatol was approximately twice more active against A549 cells, while, on the other hand, isoobtusol was approximately twice more active against RD cells. In relation to the elatol derivatives (**4**) and (**5**) and the isoobtusol derivatives (**6**) and (**8**), the modifications on the hydroxyl group at C-9 lead to a reduction in the observed cytotoxicity. Carbamate derivatives (**3**) and (**6**) were approximately three times less active than the original compounds against A549 cells, while derivative (**3**) was just slightly less active than elatol itself towards RD cells. An increase of the cytotoxic activity was observed for compound (**7**), which is the hemisuccinate of isoobtusol, since it was more cytotoxic than its precursor against A549 cells (CC_50_ = 10.74 µM) and also against RD cells (CC_50_ = 4.93 µM). It is interesting to note that the hemissuccinate derivative of isoobtusol (**7**) and isoobutusol itself (**2**) showed similar profiles being more active against RD than to A549 cells. Nevertheless, the hemisuccinate derivative of elatol (**4**) as well the sulfamate derivatives (**5**) and (**8**) were less active than the original compounds to both cell lines. These findings suggest that the substituent at C-9 plays an important role for the cytotoxic activity against these cell lines, and the best results were observed with the free hydroxyl or the hemisuccinate group. 

## 3. Experimental

### 3.1. General

NMR spectra were recorded in CDCl_3_ or CD_3_OD at 500.13 and 125.13 MHz for ^1^H and ^13^C, respectively, on a Bruker Avance 2500 MHz NMR spectrometer with TMS or the signal of residual non-deuterated solvent as internal standard. High-resolution ESI (ESI-HR-MS) mass spectra were recorded on a Bruker-Daltronics MicroTOF-Q II mass spectrometer. IR spectra were obtained on a Shimadzu Prestige 2 instrument by using KBr pellets. Column chromatography was performed using silica gel (200–300 mesh, Merck^®^). TLC was carried out using Kieselgel 60 F_254_ (Merck^®^ aluminum support plates). All solvents were of analytical grade and were purchased from Nuclear^®^. Chemical reagents, as well as deuterated solvents for NMR experiments, were purchased from Aldrich Chemical Company (Milwaukee, Wisconsin, USA).

### 3.2. Algal Material

*Laurencia microcladia* was collected by hand in March 2010 at the lower intertidal zone of Praia da Sepultura (27°07′54′′S and 48°31′40′′W), Santa Catarina, Southern Brazilian coast. Voucher samples are kept at the Herbarium of the Department of Botany, Federal University of Santa Catarina (FLOR 14516–14520). 

Fresh material of *L. microcladia* (1 kg) was exhaustively extracted with ethanol at room temperature for 3 days (three times). The concentrated extract (5 g) was partitioned with ethyl acetate and water, affording 2 g of aqueous fraction and 3 g of ethyl acetate fraction. This latter was fractionated as previously described by Lhullhier *et al.* [8] to yield pure compounds **1** (500 mg) and **2** (400 mg).

### 3.3. Synthesis

#### 3.3.1. Elatol 9-Carbamate (**3**)

To a solution of **1** (100 mg, 0.3 mmol) in CHCl_3_ (0.4 mL) chlorosulfonyl isocyanate was added dropwise (0.08 mL, 0.9 mmol) at 20 °C under nitrogen atmosphere with vigorous stirring. The reaction mixture was left overnight during 16 h, then, a solution of THF/H_2_O (1/1 v/v, 2 mL) was added, followed by a saturated solution of NaHCO_3_ (1 mL). The obtained suspension was stirred for an additional 2 h at 20 °C and then diluted with chloroform (10 mL). The organic phase was separated and washed with H_2_O (2 × 10 mL), dried over anhydrous sodium sulphate and evaporated under reduced pressure. The resulting crude product was purified by column chromatography on silica gel using hexane/EtOAc (8:2) and afforded 80 mg (71%) of carbamate 3 as a white solid. Mp: 79–81 °C; IR (KBr): 3470, 3240, 1705, 1600, 1385, 1334, 1078, 1055 cm^−1^, ^1^H NMR (500 MHz, CDCl_3_): δ = 1.04 (3H, s, H-12), 1.10 (3H, s, H-13), 1.66 (1H, m, H-5α), 1.70 (3H, s, H-15), 1.78 (1H, m, H-4α), 1.81 (1H, m, H-5β), 1.97 (1H, m, H-4β), 2.36 (1H, d, *J* = 17.3 Hz, H-1β), 2.51 (1H, dd, *J* = 15.0/2.8 Hz, H-8α), 2.60 (1H, d, *J* = 15.0 Hz, H-8β), 2.61 (1H, d, *J* = 17.3 Hz, H-1α), 4.53 (1H, d, *J* = 3.4 Hz, H-10), 4.77 (1H, s, H-14β), 5.04 (1H, s, H-14α), 5.15 (1H, dd, *J* = 6.3/3.4 Hz, H-9). ^13^C NMR (126 MHz, CDCl_3_): δ = 19.6 (C-15), 20.4 (C-12), 24.4 (C-13), 25.7 (C-5), 29.4 (C-4), 37.1 (C-8), 38.8 (C-1), 43.6 (C-11), 49.2 (C-6), 63.4 (C-10), 74.5 (C-9), 116.0 (C-14), 124.2 (C-2), 128.3 (C-3), 140.8 (C-7), 156.0 (C-1′); ESI-MS *m/z* 398.0496 [M + Na]^+^ (calcd for C_16_H_23_BrClNNaO_2_, 398.0493), 400.0482 [M + 2 + Na]^+^ (calcd. 400.0471), 402.0444 [M + 4 + Na]^+^ (calcd. 402.0449), observed isotopic pattern (398/400/402): 79/100/22, (theoretical: 76/100/25).

#### 3.3.2. Elatol 9-Hemisuccinate (**4**)

To a solution of **1** (50 mg, 0.15 mmol) in CH_2_Cl_2_ (1 mL) and pyridine (0.1 mL), succinic anhydride (150 mg, 1.5 mmol) and a catalytic amount of DMAP were added with stirring at 20 °C. After 24 h, the mixture was diluted with CH_2_Cl_2_ (10 mL) and washed with HCl 1 N (2 × 10 mL). The organic phase was dried with anhydrous sodium sulfate and evaporated under reduced pressure. The resulting crude product was purified by flash column chromatography on silica gel using hexane/EtOAc (8:2), to give the hemisuccinate **4** (35 mg, 69.8% yield) as yellow oil. IR (KBr): 3100, 1730, 1160 cm^−1^; ^1^H NMR (500 MHz, CDCl_3_): δ = 1.06 (3H, s, H-12), 1.10 (3H, s, H-13), 1.65 (1H, m, H-5α), 1.70 (3H, s, H-15), 1.80 (1H, m, H-4α), 1.81 (1H, m, H-5β), 1.97 (1H, m, H-4β), 2.36 (1H, d, *J* = 17.3 Hz, H-1β), 2.43 (1H, dd, *J* = 15.0/2.7 Hz, H-8α), 2.61 (1H, d, *J* = 15.0 Hz, H-8β), 2.62 (1H, d, *J* = 17.3 Hz, H-1α), 2.66 (2H, m, H-3′), 2.71 (2H, m, H-2′), 4.53 (1H, d, *J* = 3.4 Hz, H-10), 4.76 (1H, s, H-14β), 4.99 (1H, s, H-14α), 5.28 (1H, dd, *J* = 6.3/3.4 Hz, H-9). ^ 13^C NMR (126 MHz, CDCl_3_) δ = 19.4 (C-15), 20.2 (C-12), 24.2 (C-13), 25.6 (C-5), 28.8 (C-2′), 29.1 (C-3′), 29.4 (C-4), 36.6 (C-8), 38.6 (C-1), 43.3 (C-11), 48.9 (C-6), 62.8 (C-10), 74.1 (C-9), 116.0 (C-14), 124.0 (C-2), 128.1 (C-3), 140.3 (C-7), 171.1 (C-1′), 177.4 (C-4′); ESI-MS *m/z* 455.0591 [M + Na]^+^ (calcd for C_19_H_26_BrClNaO_4_, 455.0595), 457.0580 [M + 2 + Na]^+^ (calcd. 457.0574), 459.0557 [M + 4 + Na]^+^ (calcd. 459.0555), observed isotopic pattern (455/457/459): 73/100/24, (theoretical: 76/100/26).

#### 3.3.3. Elatol 9-Sulfamate (**5**)

Formic acid (0.03 mL, 0.9 mmol) was added dropwise to chlorosulfonyl isocyanate (0.08 mL, 0.9 mmol) at 0 °C with rapid stirring. Gas evolution was observed during the addition process. The resulting viscous suspension was stirred for 18 h at room temperature. The reaction mixture was cooled to 0 °C, DMA (0.2 mL) was added, and the solution was stirred for 5 min. A solution of 1 (0.3 mmol, 100 mg) in DMA (0.5 mL) was added dropwise, and the reaction was allowed to warm to 20 °C over a 1 h period. The reaction was quenched by the successive addition of EtOAc (10 mL) and brine (5 mL). The mixture was poured on EtOAc (20 mL) and water (10 mL), the organic phase was collected, and the aqueous layer was extracted with EtOAc (20 mL). The combined organic extracts were washed with brine (2 × 10 mL), dried over Na_2_SO_4_ and concentrated under reduced pressure. Purification of the residue by flash column chromatography on silica gel using hexane/EtOAc (7:3) gave the sulfamidate **5** (30.5 mg, 24% yield) as a white solid. Mp: 199–202 °C; IR (KBr): 3333, 3265, 1557, 1350, 1196, 1140, 900 cm^−1^; ^1^H NMR (500 MHz, CDCl_3_): δ = 1.02 (3H, s, H-12), 1.10 (3H, s, H-13), 1.65 (1H, m, H-5α), 1.71 (3H, s, H-15), 1.79 (1H, m, H-5β), 1.80 (1H, m, H-4α), 1.98 (1H, m, H-4β), 2.36 (1H, d, *J* = 17.3 Hz, H-1β), 2.63 (1H, d, *J* = 15.0 Hz, H-8α), 2.60 (1H, d, *J* = 17.3 Hz, H-1α), 2.86 (1H, dd, *J* = 15.0/2.8 Hz, H-8β), 4.55 (1H, d, *J* = 3.0 Hz, H-10), 4.84 (1H, s, H-14β), 4.90 (1H, dd, *J* = 6.0/3.0 Hz, H-9), 5.17 (1H, s, H-14α); ^13^C NMR (126 MHz, CDCl_3_): δ = 19.4 (C-15), 20.1 (C-12), 24.3 (C-13), 25.6 (C-5), 29.4 (C-4), 37.1 (C-8), 38.6 (C-1), 43.6 (C-11), 49.4 (C-6), 62.6 (C-10), 82.2 (C-9), 117.1 (C-14), 124.0 (C-2), 128.2 (C-3), 139.2 (C-7); ESI-MS *m/z* 429.0587 [M + NH_4_]^+^ (calcd for C_15_H_27_BrClN_2_O_3_S, 429.0608), 431.0575 [M + 2 + NH_4_]^+^ (calcd. 431.0587), 433.0565 [M + 4 + NH_4_]^+^ (calcd. 433.0563), observed isotopic pattern (429/431/433): 73/100/28, (theoretical: 73/100/29).

#### 3.3.4. Isoobtusol 9-Carbamate (**6**)

Compound **6** was similarly prepared, according to the procedure of **3**. To a solution of **2** (100 mg, 0.24 mmol) in CHCl_3_ (0.4 mL) chlorosulfonyl isocyanate (0.06 mL, 0.74 mmol) was added dropwise at 20 °C under nitrogen atmosphere with vigorous stirring. The reaction mixture was left overnight during 16 h, and then a solution of THF/H_2_O (1/1 v/v, 1.5 mL) was added, followed by a saturated solution of NaHCO_3_ (0.7 mL). The obtained suspension was stirred for an additional 2 h at 20 °C and then diluted with chloroform (10 mL). The organic phase was separated and washed with H_2_O (2 × 10 mL), dried over anhydrous sodium sulpfate and evaporated under reduced pressure. The resulting crude product was purified by column chromatography on silica gel using hexane/EtOAc (8:2) afforded 62.4 mg (57%) of carbamate **6** as white solid. Mp: 158–160 °C; IR (KBr): 3472, 3241, 1728, 1604, 1324, 1328, 1064 cm^−1^; ^1^H NMR (500 MHz, CDCl_3_): δ = 1.11 (3H, s, H-13), 1.32 (3H, s, H-12), 1.80 (1H, ddd, *J* = 14.0/6.5/3.5 Hz, H-4α), 1.85 (1H, m, H-5α), 1.92 (3H, s, H-15), 2.06 (1H, td, *J* = 14.0/3.5 Hz, H-4β), 2.24 (1H, m, H-5β), 2.41 (1H, dd, *J* = 12.2/3.4 Hz, H-8α), 2.82 (1H, dd, *J* = 15/3.7 Hz, H-1β), 2.92 (1H, t, *J* = 12.2 Hz, H-8β), 3.17 (1H, d, *J* = 15 Hz, H-1α), 4.45 (1H, m, H-2), 4.46 (1H, dd, *J* = 3.4/1.9 Hz, H-10), 4.73 (1H, m, H-9), 4.98 (1H, s, H-14), 5.21 (1H, s, H-14); ^13^C NMR (126 MHz, CDCl_3_): δ = 24.6 (C-12), 25.1 (C-13), 25.6 (C-4), 33.1 (C-15), 33.3 (C-5), 34.0 (C-1), 35.4 (C-8), 43.7 (C-6), 44.1 (C-11), 65.2 (C-2), 67.3 (C-10), 71.1 (C-3), 72.0 (C-9), 114.6 (C-14), 146.9 (C-7), 155.6 (C-1′); ESI-MS *m/z* 477.9773 [M + Na]^+^ (calcd for C_16_H_24_Br_2_ClNNaO_2_, 477.9754), 479.9736 [M + 2 + Na]^+^ (calcd. 479.9733), 481.9727 [M + 4 + Na]^+^ (calcd. 481.9712), 483.9684 [M + 6 + Na]^+^ (calcd. 483.9692), observed isotopic pattern (477/479/481/483): 43/100/69/13, (theoretical: 44/100/70/14).

#### 3.3.5. Isoobtusol 9-Hemisuccinate (**7**)

Compound **7** was prepared in a similar way as **4**. To a solution of **2** (50 mg, 0.12 mmol) in CH_2_Cl_2_ (1 mL) and pyridine (0.1 mL), succinic anhydride (121 mg, 1.2 mmol) and a catalytic amount of DMAP were added with stirring at 20 °C. After 24 h, the mixture was diluted with CH_2_Cl_2_ (10 mL) and washed with HCl 1 N (2 × 10 mL). The organic phase was dried with anhydrous sodium sulfate and evaporated under reduced pressure. The resulting crude product was purified by flash column chromatography on silica gel using hexane/EtOAc (8:2), to give the hemisuccinate **7** (31 mg, 62% yield) as with solid. Mp: 116–118 °C; IR (KBr): 3100, 1738, 1184 cm^−1^; ^1^H NMR (500 MHz CDCl_3_:MeOD, 2:1): δ = 5.21 (1H, s, H-14α), 5.0 (1H, s, H-14β), 4.85 (1H, m, H-9), 4.46 (1H, br s, H-2), 4.42 (1H, dd, *J* = 3.4/1.9 Hz, H-10), 3.16 (1H, d, *J* = 15 Hz, H-1α), 2.95 (1H, t, *J* = 12.2 Hz, H-8β), 2.81(1H, dd, *J* = 15/3.7 Hz, H-1β), 2.67 (2H, overlapped, H-3′), 2.62 (2H, overlapped, H-2′), 2.41 (1H, m, H-8α), 2.24 (1H, m, H-5β), 2.07 (1H, td, *J* = 14.0/3.5 Hz, H-4β), 1.85 (1H, m, H-5α), 1.82 (1H, m, H-4α), 1.31 (3H, s, H-12), 1.11 (3H, s, H-13); ^13^C NMR (126 MHz, CDCl_3_:MeOD, 2:1): δ = 174.7 (C-4′), 172.0 (C-1′), 146.8 (C-7), 114.9 (C-14), 72.0 (C-9), 71.2 (C-3), 66.3 (C-10), 65.3 (C-2), 43.9 (C-6), 35.2 (C-8), 34.1 (C-1), 33.4 (C-5), 33.1 (C-15), 29.0 (C-3′), 28.9 (C-2′), 25.7 (C-4), 25.1 (C-13), 24.6 (C-12); ESI-MS *m/z* 530.0295 [M + NH_4_]^+^ (calcd for C_19_H_31_Br_2_ClNO_4_, 530.0303), 532.0276 [M + 2 + NH_4_]^+^ (calcd. 532.0282), 534.0260 [M + 4 + NH_4_]^+^ (calcd. 534.0262), 536.0175 [M + 6 + NH_4_]^+^ (calcd. 536.0244), observed isotopic pattern (530/532/534/536): 42/100/74/11, (theoretical: 43/100/72/15).

#### 3.3.6. Isoobtusol 9-Sulfamate (**8**)

Compound **8** was prepared according to the procedure of **5**. Formic acid (0.03 mL, 0.73 mmol) was added dropwise to chlorosulfonyl isocyanate (0.06 mL, 0.73 mmol) at 0 °C with rapid stirring. Gas evolution was observed during the addition process. The resulting viscous suspension was stirred for 18 h at room temperature. The reaction mixture was cooled to 0 °C, DMA (0.2 mL) was added, and the solution was stirred for 5 min. A solution of **2** (100 mg, 0.24 mmol) in DMA (0.3 mL) was added dropwise, and the reaction was allowed to warm to 20 °C over a 1 h period. The reaction was quenched by the successive addition of EtOAc (10 mL) and brine (5 mL). The mixture was poured on EtOAc (20 mL) and water (10 mL), the organic phase was collected, and the aqueous layer was extracted with EtOAc (20 mL). The combined organic extracts were washed with brine (2 × 10 mL), dried over Na_2_SO_4_ and concentrated under reduced pressure. Purification of the residue by flash column chromatography on silica gel using hexanes/EtOAc (7:3) gave the sulfamidate **8** (15.0 mg, 13% yield) as a white solid. Mp: 143–145 °C; IR (KBr): 3410, 3282, 1520, 1366, 1182, 1167, 933 cm^−1^; ^1^H NMR (500 MHz, CDCl_3_): *δ* = 5.26 (1H, s, H-14α), 5.03 (1H, s, H-14β), 4.60 (1H, m, H-9), 4.45 (1H, m, H-2), 4.44 (1H, dd, *J *= 4.0/2.0 Hz, H-10), 3.16 (1H, d, *J *= 15 Hz, H-1α), 3.04 (1H, t, *J *= 12.5 Hz, H-8β), 2.82 (1H, dd, *J *= 15.6/3.2 Hz, H-1β), 2.58 (1H, dd, *J *= 12.5/2.0 Hz, H-8α), 2.23 (1H, m, H-5β), 2.07 (1H, dt, *J* = 14.0/2.0 Hz, H-4β), 1.86 (1H, m, H-5α), 1.80 (1H, m, H-4α), 1.34 (3H, s, H-12), 1.10 (3H, s, H-13); ^13^C NMR (126 MHz, CDCl_3_): *δ* = 146.5 (C-7), 115.5 (C-14), 77.9 (C-9), 70.9 (C-3), 66.3 (C-10), 65.2 (C-2), 44.0 (C-6), 36.2 (C-8), 34.1 (C-1), 33.3 (C-5), 33.1 (C-15), 25.7 (C-4), 25.0 (C-13), 24.7 (C-12); ESI-MS *m/z* 513.9422 [M + Na]^+^ (calcd for C_15_H_24_Br_2_ClNNaO_3_S, 513.9424), 515.9419 [M + 2 + Na]^+^ (calcd. 515.9403), 517.9378 [M + 4 + Na]^+^ (calcd. 517.9381), 519.9355 [M + 6 + Na]^+^ (calcd. 519.9358), observed isotopic pattern (513/515/517/519): 73/100/28/2, (theoretical: 73/100/29/2).

### 3.4. Cell Lines

The human embryo rhabdomyosarcome cells (RD) were obtained from Adolfo Lutz Institute, São Paulo, SP, Brazil. The human non-small cell lung cancer (A549 cells) were kindly provided by Dr. Rosina Gironès from the Microbiology Department of the University of Barcelona, Spain.

### 3.5. MTT Assay

RD and A549 cells were grown in minimal essential medium (MEM, Cultilab, São Paulo, Brazil). Both cell lines were supplemented with 10% fetal bovine serum, and 100 U/mL penicillin G, 100 μg/mL streptomycin, and 25 μg/mL amphotericin B (Gibco, São Paulo, Brazil). Cell cultures were kept in tissue culture flasks in a humidified atmosphere of 5% CO_2_ at 37 °C. The effect of the samples treatment on proliferation of RD and A549 cells was measured by the MTT [3-(4,5-dimethylthiazol-2-yl)-2,5-diphenyl tetrazolium bromide] assay [22]. Approximately 104 cells were plated per well in 96-well plates and treated with different concentrations of each sample. After 48 h at 37 °C, the medium was removed, 50 μL of MTT reagent (1 mg/mL) were added to each well, and cells were further incubated at 37 °C for more 4 h. The MTT solution was removed, 100 μL of dimethyl sulfoxide (Nuclear, Brazil) were added to each well to dissolve formazan crystals, and the plates were gently shaken, whereby crystals were completely dissolved. The absorbances were read on a multiwell spectrophotometer (Tecan, Grödig, Austria) at 540 nm. The 50% cytotoxic concentration (CC 50) of each sample was defined as the concentration that reduced cell viability by 50% when compared to untreated controls. Paclitaxel (0 to 10 μM, Glenmark, Brazil) was used as positive control (purity > 98%).

### 3.6. Statistical Analysis

The mean ± standard deviations are representative of three independent experiments. For determination of CC 50 values non-linear regressions of concentration-response curves were used.

## 4. Conclusions

The preparation and characterization of new derivatives of elatol and isoobtusol was described as well as their *in vitro* inhibitory effects on two human tumor cell lines growth. The obtained results will stimulate the introduction of further structural modifications in these natural products in order to enhance these effects.
